# Eco-Friendly Paper-Based Electrochemical Device Manufactured with Low-Cost School Supplies and Carbon Black-Graphene Ink for Determination of Endocrine Disruptor in Food

**DOI:** 10.1021/acsomega.6c02401

**Published:** 2026-06-30

**Authors:** Alexsandra Dias da Silva, Jéssica Rocha Camargo, Rodrigo Silva de Oliveira, Bruno Campos Janegitz, Tiago Almeida Silva

**Affiliations:** † Department of Chemistry, 28120 Federal University of Viçosa, 36570-900 Viçosa, Brazil; ‡ Laboratory of Sensors, Nanomedicine, and Nanostructured Materials, 67828 Federal University of São Carlos, 13600-970 Araras, Brazil

## Abstract

A new paper-based analytical device (PAD) was developed using paper and shellac waterproofing, manufactured with a carbon black (CB) and graphene (GP) ink. The content of the ink components was evaluated and optimized for the sensitive voltammetric detection of the endocrine disruptor bisphenol A (BPA). Scanning electron microscopy (SEM), X-ray diffraction (XRD) and Fourier-Transform Infrared Spectroscopy (FT-IR) characterizations were carried out. Enhanced electron transfer kinetic was observed and higher analytical signal was recorded with CB-GP-based PADs for the irreversible oxidation of BPA. When applying square-wave voltammetry under optimized conditions, the 5GP-CB-PAD operated in a linear working range of 5.1 × 10^–6^ to 5.0 × 10^–4^ mol L^–1^ for BPA, with limits of detection (LOD) and quantification (LOQ) of 1.7 × 10^–6^ mol L^–1^ and 5.1 × 10^–6^ mol L^–1^, respectively. When applied to canned corn water samples, recoveries of over 84% were recorded, demonstrating the possibility of applying it to this food sample. The new sensor could contribute to the development of other devices made from alternative and sustainable materials.

## Graphical abstract

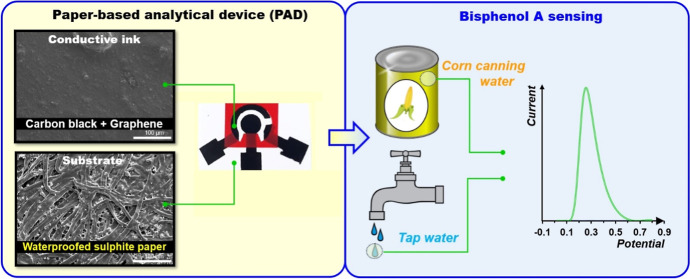

## 1 Introduction

Progress in the development of paper-based analytical devices (PADs) stems from the many advantages that paper brings to proposed projects. Among the possible papers that can be delivered (cellulose paper; Whatman No. 1, 2 and 3 filter papers; office paper; handmade paper; tree leaves; and Toray paper), sulfite paper has advantages that include easy sourcing and cost-effectiveness.^1^ In addition, PADs have advantages over classic electrodes: they are flexible, biocompatible, highly reproducible, can be disposed of by incineration and have high portability for in situ detection.^2,3^ PADs can be combined with different detection methods, where electrochemical detection offers benefits such as easy miniaturization of the electrochemical components and appropriate sensitivity and selectivity of the method.^4^

In addition to the substrate, PADs require a conductive ink.^5,6^ This conductive ink can be formulated with varying concentrations of different conductive materials, such as macromolecules and nanoparticles, phthalocyanine-based catalysts, metal oxides and others.^7–12^ Carbon black (CB) is a carbonaceous nanomaterial that has electrical conductive and electrocatalytic properties and can disperse in different solvents.^5,6,13^ CB is an easily obtainable material that possesses a larger active surface area, high π–π interactions, ease of modification, and interaction with other biomaterials, and features numerous defect sites that enable effective electron transfer in electrochemical sensing applications.^14–16^ Furthermore, graphene (GP) has strong bonds between the carbon atoms, making the material highly stable from a mechanical and thermal point of view and very low electrical resistance.^17,18^ Ink formulations based on graphene and its derivatives have already been reported in the literature.^19^ The combination of CB and graphene in the formulation of conductive inks for a PAD-type device shows the different possibilities in the development of (bio)­sensors with unique characteristics in the field of electroanalytical chemistry.

Bisphenol A (2,2-bis (4-hydroxyphenyl) propane) (BPA) is a compound widely used in containers, toys, plastic food packaging, resins and dental materials.^20,21^ It is estimated that by 2050, global production of BPA will be approximately 590 billion kilograms.^22^ Time–temperature factors can lead to BPA leaching from plastic products and contaminating food, drink and water, which can cause health problems in humans and animals. BPA has a structure that resembles the structure of the hormones estradiol and diethylstilbestrol.^23–25^ This, in considerable quantities, causes it to act as an endocrine disruptor, with effects on the development of type 2 diabetes, obesity problems and cases of cancer.^26^ The European Food Safety Authority (EFSA) and the US Environmental Protection Agency (EPA) have established maximum exposure levels for BPA. The EFSA has established a tolerable daily intake (TDI) of 50 μg kg^–1^ of body weight, while the European Union (EU) has implemented specific migration limits (SML) for BPA in plastic materials and food packaging coatings.^27^ The study by Zhao et al. found that concentrations of BPA ranging from 1.1 to 12.8 mg L^–1^ (4.8 μmol L^–1^ to 56.1 μmol L^–1^) were considered toxic to various groups of organisms, including daphnia, mysids and freshwater and saltwater fish.^22^

In this context, there is a growing need today for rapid BPA detection with minimal sample preparation and using a sensor made from low-cost materials, enabling the development of on-site, disposable sensors. Sensors designed to detect BPA typically employ nanoparticles and/or metal oxides and involve numerous sensor fabrication steps. Thus, this study aimed to develop a disposable sensor using school supplies, based on active printing devices (PADs) for BPA detection, manufactured with carbon black conductive ink and graphene. To the best of our knowledge, this is the first report on PADs made from sulfite paper and carbon black conductive ink and graphene as the main components of a disposable BPA sensor.

## 2 Experimental

### 2.1 Reagents, Materials and Solutions

The reagents used to prepare the phosphate buffer solutions and the BPA stock solution were purchased from Sigma-Aldrich, Synth and/or Fluka, all in analytical grade. A colorless shellac binder (Acrilex) was purchased in the local market. Vulcan XC72R carbon black was kindly provided by the Cabot Corporation. GF powder was purchased from Fisher Chemical (specifications: carbon content >95%, average specific area of 100–140 m^2^ g^–1^ and average lateral size of 1–2 μm). All reagents were used as received, without additional purification steps. The aqueous solutions were prepared with ultrapure water (ρ > 18.2 MΩ cm) obtained from the Milli-Q purification system.

### 2.2 Preparation of the Conductive Ink

The conductive ink was prepared using a fixed composition of colorless shellac binder (87% w/w) and varying levels of CB (13%, 12%, 10%, and 8% w/w) and graphene (0%, 1%, 3%, and 5% w/w) as conductive solid materials. A double asymmetric centrifuge (SpeedMixerTM Dac 150.1 FVZ-K, FlackTec Inc.) was used to mix the inks in two stages at 3500 rpm for 3 min. After centrifugation, the inks were ready for use. A graphene-free ink (i.e., composed of only CB) was also prepared for comparison purposes.

### 2.3 Making the PAD Sensors

A waterproofing agent (Ultra lub) (composition: fluorocarbon resin, organic solvents and butane/propane propellant) was applied to sulfite paper (Report Suzano, 75 g m^–2^) to prevent absorption of the measurement solutions by the paper, and this was the substrate used for the screen printing. The electrode set was designed and its mold was made from adhesive paper masks (Colacril). The Silhouette Studio software and the Silhouette Cameo 3 cutting printer were used to cut out the masks. The blank masks were attached to the waterproofed substrate and the freshly prepared CB-GP ink was spread over the surface using a spatula. The mask mold was removed after immediately applying the ink, resulting in the electrodes being inked onto the chosen substrate. Due to the quick drying time of the shellac, the electrodes were dried after just a few minutes. Finally, the electrodes were cut out individually and a mask of adhesive paper was applied to the electrodes to delimit the geometric area of analysis. After this process, the electrodes were ready for use and were stored under vacuum to preserve them. Figure 1 shows a diagram with the followed steps of the PADs preparation.

**Figure 1:**
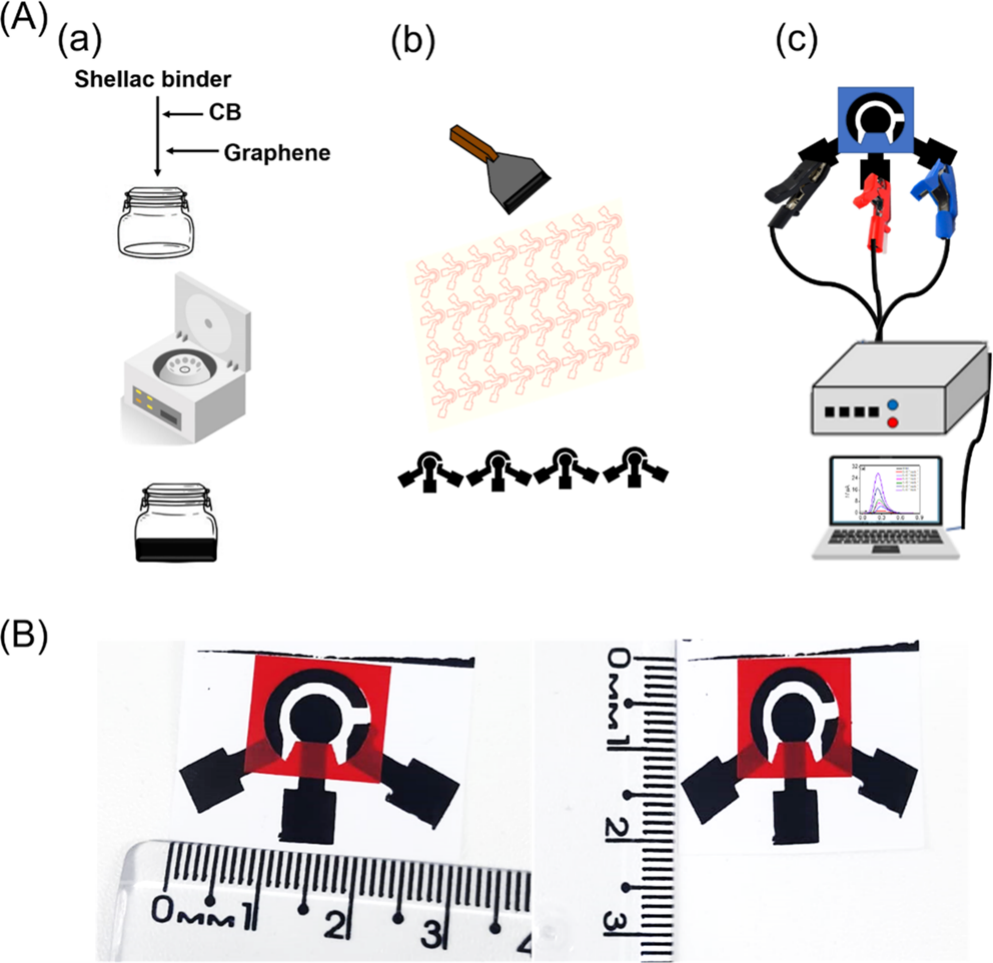
(A) Followed steps for production of the proposed PADs. (a) Obtaining the conductive ink based on CB-GP. (b) Screen-printing of the formulated ink on the electrode molds. (c) PAD is ready and connected to the potentiostat for use. (B) Real images of the developed sensor.

### 2.4 Characterizations

For the physicochemical characterizations, X-ray diffractograms (XRD) were obtained under scanning conditions in the angular range of 2θ of 2° to 90°, with a resolution of 0.02° min^–1^, angular step of 10° min^–1^, CuKα radiation (λ = 1.5418 Å) voltage of 45 kV and anode current of 40 mA, carried out on the MiniFlex600, Rigaku. The chemical structure and functional groups of the samples were characterized by Fourier Transform Infrared Spectroscopy (FT-IR) using a TENSOR II spectrophotometer (Bruker, Germany). The spectra were recorded in KBr pellet mode within the spectral range of 4000–400 cm^–1^, with a resolution of 4 cm^–1^ and 64 accumulated scans per sample. The FT-IR analysis was employed to identify the main functional groups and assess possible chemical interactions between the constituents of the materials.

Scanning electron microscopy (SEM) images were obtained using a ThermoFisher Scientific Prisma E scanning electron microscope, with a voltage acceleration of 10 kV and low vacuum mode of 50 Pa. The electrodes were attached to aluminum SEM pins using a carbon strip. The samples were first metalized with gold. Micrographs were acquired at different magnifications to evaluate the surface morphology, particle distribution, and microstructural features of the materials. The obtained SEM images were further analyzed using the ImageJ software (National Institutes of Health, USA) to estimate the particle size of the graphene samples. Image calibration was performed using the scale bar provided in each micrograph, and multiple particles were measured to obtain representative size distributions. Contact angle analysis was performed by applying 50 μL of ultrapure water to the working papers and working electrodes of the PAD devices.

### 2.5 Electrochemical Measurements

The electrochemical assays were carried out in a PGSTAT 101 Metrohm potentiostat/galvanostat (Eco Chemie), managed by Nova 2.1.7 software. The cyclic voltammetry (CV) technique was used to electrochemically characterize the produced PADs. In this case, a 1.0 mmol L^–1^ ferrocenemethanol (FCN) solution was used as the electrochemical probe.

The square-wave voltammetry (SWV) technique was used for optimization studies and the detection of BPA. Aqueous solutions of 0.1 mol L^–1^ KCl and 0.2 mol L^–1^ phosphate buffer (PB, at pH 8.0) were used in different analyses as the respective supporting electrolytes.

### 2.6 Samples Analysis

After evaluating the optimum PADs and optimizing the SWV parameters, studies were carried out on the recovery of BPA in tap water and canned green corn water samples. Tap water and canned corn water were used to prepare the supporting electrolyte, 0.2 mol L^–1^ phosphate buffer at pH 8.0, and to prepare the BPA test solutions. The canned water sample was obtained from a plastic package of green corn purchased from a local market in the city of Araras-SP, and a dilution of 2× (v/v) (water from the plastic packet: ultrapure water) was made.

## 3 Results and Discussion

### 3.1 Physicochemical Characterization of the PAD

SEM images were taken to characterize the substrate and the designed PADs. Figure 2a–d) display SEM images of the sulfite paper before and after the application of the waterproofing agent, respectively. In both cases, a homogeneous and rough surface can be seen, with fibrous lines along its entire length. Mapping by energy-dispersive X-ray spectroscopy (EDS) enabled the chemical elements present in the paper without and with waterproofing to be elucidated (Figure S1a,b). The EDS of the paper without waterproofing (Figure S1c) shows the presence of C (35.2%), O (61.4%) and Ca (3.4%), where the element Ca may have been incorporated into the paper at one of its manufacturing stages.^28^ On the other hand, the EDS of the waterproofed paper (Figure S1d) shows the presence of C (39.7%), O (40.8%), Ca (2.0%) and F (17.5%), where element F may have its origin in the waterproofing agent chosen to protect the paper. The chemical elements identified, as well as the proportions indicated for each one, may vary depending on how the paper is manufactured and the reagents used.

**Figure 2:**
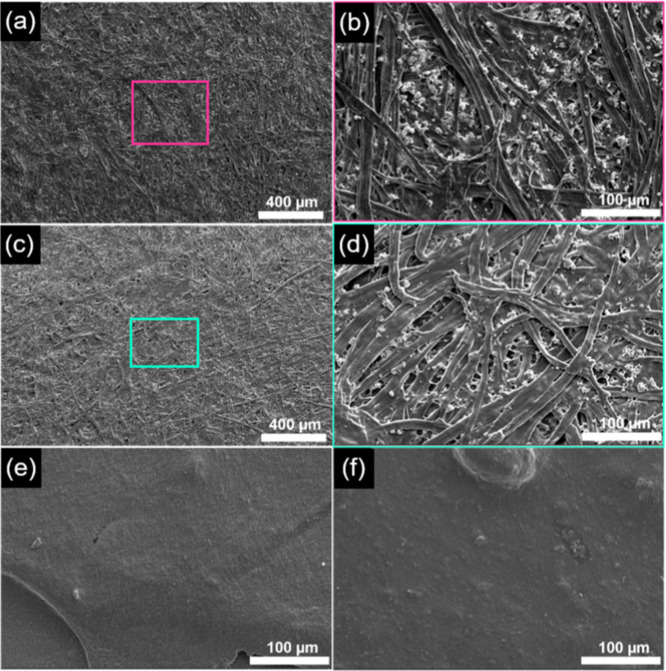
SEM images of sulfite paper (a,b) before and (c,d) after the application of the waterproofing agent. (e) CB-PAD sensor’ surface and (f) 5GP-CB-PAD sensor’ surface.

The contact angle wettability analysis was carried out on the paper in the presence and absence of the waterproofing agent, as well as on the complete PAD. The images are shown in Figure S2. The waterproofed paper showed a contact angle of between 150° and 90° (Figure S2b), characteristic of a hydrophobic surface. The nonwaterproofed paper (89° angle) has a hydrophilic surface (Figure S2a). These data prove the success of waterproofing the sulfite paper used. The deposition of the conductive ink (Figure S2c,d) favored a hydrophilic surface, which can be explained by the presence of polar functional groups in the CB and CB-GF.^29^ The SEM images in Figure 2e,f show the surface morphology of CB-PAD and 5GP-CB-PAD (PAD prepared with conductive ink containing 5% (m/m) graphene), respectively. Irregular surfaces can be seen. Finally, more magnified SEM images of the CB-PAD sensor (Figure 3a,b) and 5 GP-CB-PAD (Figure 3c,d) were also recorded and the thickness of the deposited inks were identified as 68.13 and 83.25 μm for CB-PAD and 5GP-CB-PAD, respectively.

**Figure 3:**
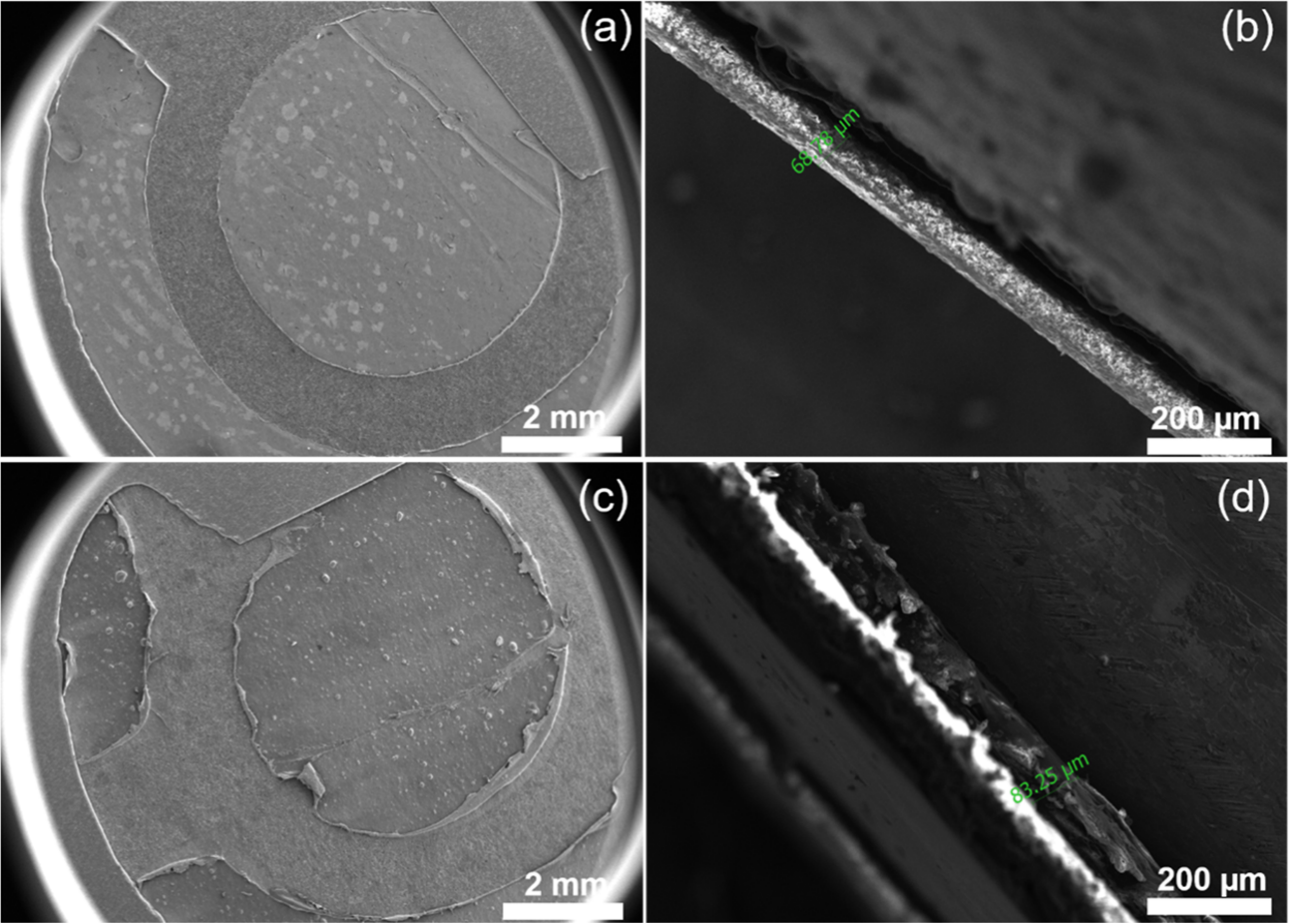
SEM images recorded for CB-PAD (a) 19× and (b) 200×, and for 5GP-CB-PAD (c) 19× and (d) 200×.

XRD analyses were carried out to understand the structures of the samples used in the manufacture of PADs. Figure 4a–d provide the diffractograms of the sulfite paper subjected to different treatments: (a) without applying waterproofing, (b) without waterproofing and with shellac deposition, (c) with waterproofing applied and without shellac deposition and (d) with waterproofing and shellac deposition. In general, well-defined peaks were observed in the diffractograms in the 200 plane at 2θ = 15.7° and 2θ = 21.8°, attributed to the paper sheet. The definition and intensity of the peaks varies according to the sheet treatment temperature.^30^ Similar standards for cellulose studies have been reported by other authors.^31,32^ Although in the literature it has already been reported that the XRD of carbon black shows peaks in the 2θ range at 20°–30° and peaks around 2θ at 40°–50°,^33,34^ these peaks were not observed in the samples analyzed (Figure 4e–g shows the graphene diffractogram where the graphite peak is observed in the 002 plane (2θ = 26.5°) and the 004 plane (2θ = 55.5°). These characteristic points have also been identified by other authors.^35^ The same peaks are seen in the 5GP-CB-PAD sensor (Figure 4h).

**Figure 4:**
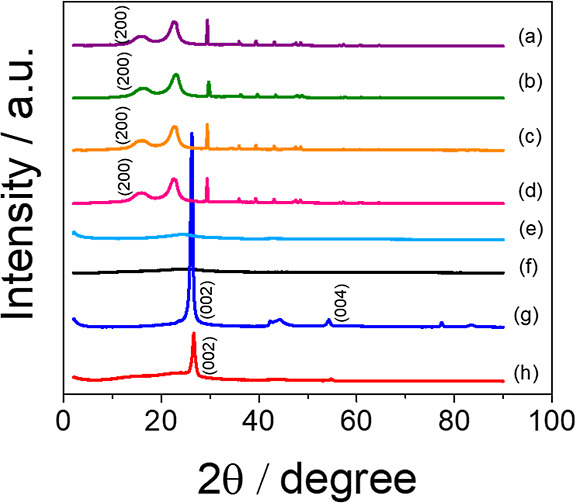
XRD patterns of (a) paper without the waterproofing agent, (b) paper without waterproofing agent and with shellac deposition, (c) paper with the waterproofing agent and without shellac deposition, (d) paper with waterproofing agent and shellac deposition, (e) carbon black powder, (f) CB-PAD sensor, (g) graphene powder and (h) 5GP-CB-PAD sensor.

The FT-IR spectra revealed clear differences between graphene (GP) and carbon black (CB), providing additional evidence of the distinct structural organization of graphene (Figure 5). Both materials exhibited a broad absorption band around 3400 cm^–1^, assigned to O–H stretching vibrations from hydroxyl groups and adsorbed water. However, the graphene spectrum displayed a characteristic band at 1578 cm^–1^, attributed to the stretching vibration of conjugated sp^2^ C=C bonds within the graphitic lattice. This band was shifted relative to the CB spectrum, which exhibited a band at 1432 cm^–1^, commonly associated with less ordered aromatic domains and oxygen-containing functional groups. The higher wavenumber observed for graphene indicates a greater degree of graphitic ordering and extended π-conjugation. Additional differences were observed in the 1000–1100 cm^–1^ region, where graphene exhibited a band at 1093 cm^–1^, while CB presented a band at 1031 cm^–1^. These absorptions are generally assigned to C–O and epoxy-related vibrations, suggesting distinct surface chemical environments and a lower concentration of oxygenated defects in graphene.

**Figure 5:**
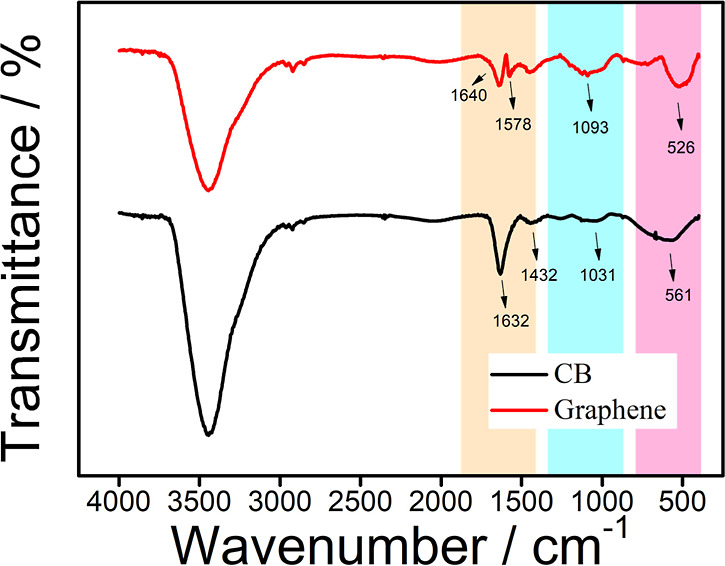
FT-IR spectra of graphene (GP) and carbon black (CB).

Furthermore, the lower intensity of oxygen-containing functional group bands in graphene compared to CB is consistent with a more ordered carbon framework and a higher graphitization degree. Taken together, the FT-IR results corroborate the XRD findings and support the successful identification of graphene through the presence of a highly conjugated sp^2^ carbon network and reduced structural disorder relative to carbon black. While Raman spectroscopy and XPS would indeed provide additional information regarding defect density, graphitization degree, and surface elemental composition, these analyses were not available during the present study. Nevertheless, the combined XRD and FT-IR results provide consistent evidence supporting the structural distinction between graphene and CB.

Particle/agglomerate dimensions were determined from binarized SEM images using the Feret diameter as the characteristic size parameter (Figure 6a–c). The ImageJ analysis identified individual graphene agglomerates, yielding a mean Feret diameter of 4.59 ± 3.52 μm, with particle sizes ranging from 1.41 to 11.48 μm. The median Feret diameter was 2.88 μm, indicating the presence of a population of small agglomerates together with larger aggregates. At 500× magnification (Figure 6a), the graphene powder exhibits a highly aggregated morphology, forming clusters distributed throughout the observed field. Quantitative analysis revealed agglomerates predominantly within the 1–12 μm size range, with larger clusters reaching approximately 11.5 μm. This broad particle size distribution is consistent with the strong van der Waals interactions commonly reported for graphene-based materials, which promote restacking and aggregation. At 5000× magnification (Figure 6b), the higher-resolution image confirms that the larger aggregates observed at low magnification are composed of smaller irregular graphene domains with rough surfaces and nonspherical morphologies.

**Figure 6:**
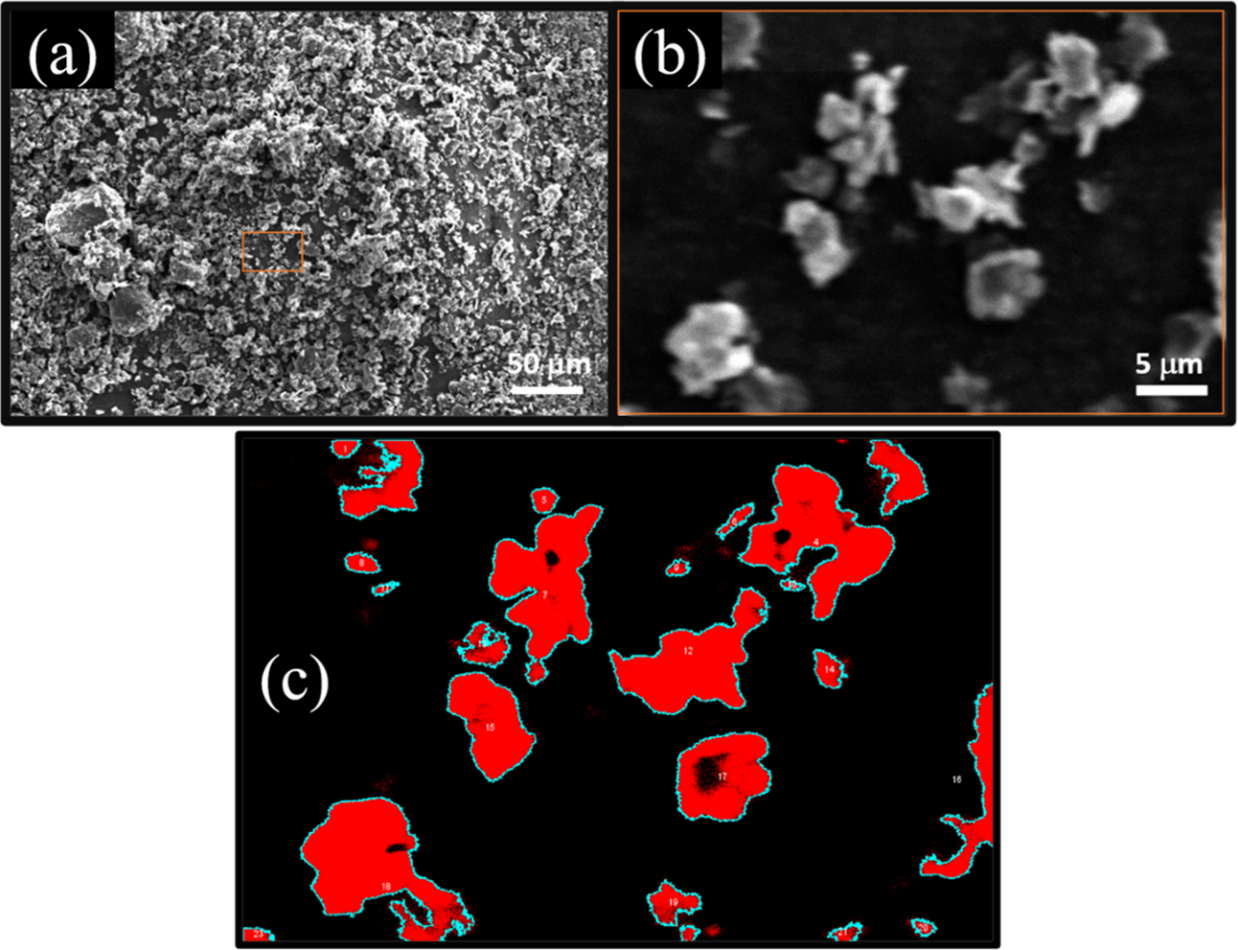
SEM micrographs of graphene at (a) 500× and (b) 5000×. (c) ImageJ-based quantitative analysis of the SEM micrographs.

The calculated average aspect ratio (AR = 2.38 ± 2.55) and roundness (0.57 ± 0.20) indicate elongated and irregularly shaped particles rather than compact spherical structures. Furthermore, the average solidity value (0.61 ± 0.18) suggests the presence of surface irregularities and edge defects, which are characteristic of exfoliated graphitic materials. The inclusion of this quantitative analysis strengthens the SEM discussion by providing objective morphological descriptors rather than relying solely on qualitative observations. These results corroborate the structural characterization obtained from XRD and FT-IR and support the presence of graphene aggregates with micrometric dimensions and irregular morphology.

### 3.2 Electrochemical Characterization

To investigate the use of a conductive ink made of graphene, carbon black and shellac binder deposited on a sulfite paper substrate in the manufacture of PADs, electrochemical measurements were carried out using the FCN redox probe. An ink without graphene was also tested for comparison. Figure 7 shows the cyclic voltammograms (CVs) recorded of the PADs prepared with conductive ink without graphene (CB-PAD) and containing graphene in its composition at different levels: 1% m/m (1GP-CB-PAD), 3% m/m (3GP-CB-PAD) and 5% m/m (5GP-CB-PAD), respectively.

**Figure 7:**
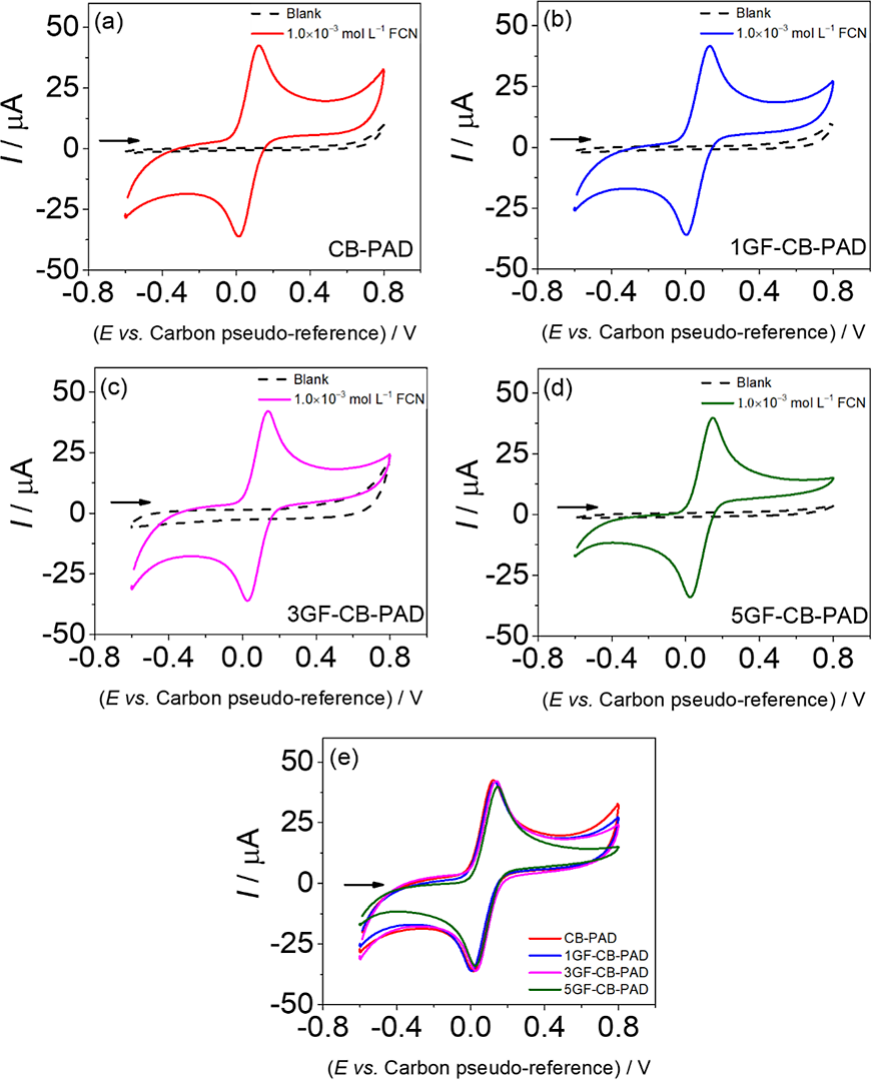
Cyclic voltammograms recorded in 0.1 mol L^–1^ KCl in the absence (blank solution) and presence of 1.0 × 10^–3^ mol L^–1^ FCN using the PADs manufactured with conductive inks containing (a) 0% (CB-PAD), (b) 1% (1GP-CB-PAD), (c) 3% (3GP-CB-PAD) and (d) 5% m/m (5GP-CB-PAD) of graphene. *v* = 50 mV s^–1^. (e) Comparison of the obtained cyclic voltammograms in the presence of FCN using the different produced PADs.

Using the four PADs tested, it was possible to observe a well-defined peak shape voltammogram for the formation of the oxidation–reduction redox pair of FCN.

Figure 7e shows a comparison of the voltammetric response of the PADs in the presence of the FCN redox probe. The intensity of the anodic peak current remained constant as the percentage of graphene in the ink increased. However, the peak-to-peak potential difference between the anodic (*E*_pa_) and cathodic (*E*_pc_) (Δ*E*_p_ = *E*_pa_–*E*_pc_) decreased from 112.30, 126.96, 95.21, and 87.89 mV in the PADs developed with ink containing 0% to 5% GP, respectively. This result suggests higher conductivity of the PAD with 5% w/w graphene in the ink formulation and enhanced heterogeneous electron transfer rate constant (*k* ^0^). Therefore, since it exhibits the highest reversibility factor among the evaluated inks, the PAD sensor fabricated with 5% w/w graphene in the conductive ink was used in subsequent studies.

To obtain information on the electrochemical behavior of the PADs produced, the CB-PAD and 5GP-CB-PAD, Figure 8a and c, respectively, were subjected to CV at different scan rates from 10 to 100 mV s^–1^ in 0.1 mol L^–1^ of KCl with 1 mmol L^–1^ of FCN.

**Figure 8:**
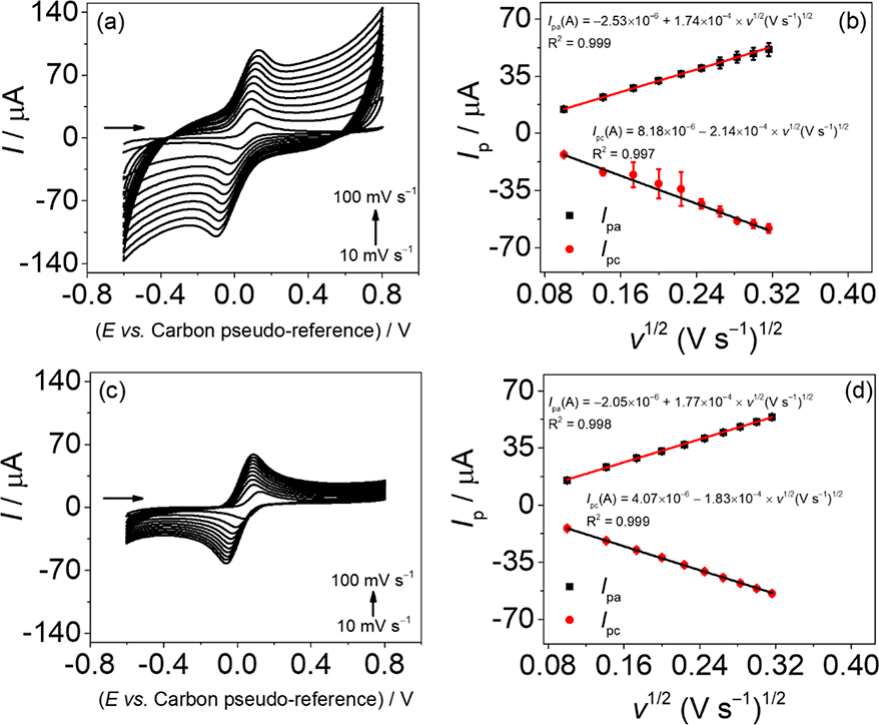
Cyclic voltammograms recorded at different scan rates (10–100 mV s^–1^) in 0.1 mol L^–1^ KCl in the presence of 1.0 × 10^–3^ mol L^–1^ FCN using the PADs manufactured with conductive inks containing (a) 0% (CB-PAD) and (c) 5% m/m of graphene (5GP-CB-PAD). Plots of *I*_p_ vs *v* ^1/2^ obtained for the PADs manufactured with conductive inks containing (b) 0% (CB-PAD) and (d) 5% m/m of graphene (5GP-CB-PAD).

From the CVs shown in Figure 8a and c, it was observed that the anodic and cathodic peak currents were directly proportional to the square root of the scan rate (*I*_p_ vs *v* ^1/2^ plots), shown in Figure 8b for the CB-PAD and Figure 8d for the 5GP-CB-PAD sensor. The linear relationship observed between *I*_pa_ vs *v* ^1/2^ and *I*_pc_ vs *v* ^1/2^, *R* ^2^ > 0.997, agrees with the relationship predicted by the Randles-Ševčík equation for redox processes controlled by diffusion mass transport. From this, the electroactive surface area and heterogeneous electron transfer rate constant of each PAD was predicted.

The electroactive surface area was obtained from Randles-Ševčík equation, displayed in eq 1

1 $$I_{p}=\pm \left(2.69\times 10^{5}\right)n^{3/2}AD^{1/2}cv^{1/2}$$

where *I*_p_ is the anodic or cathodic peak current, *n* is the number of electrons involved in the redox process (*n* = 1), *A* is the electroactive area (in cm^2^), *D* is the diffusion coefficient of the electroactive species (*D* = 7.6 × 10^–6^ cm^2^ s^–1^ for FCN in 0.1 mol L^–1^ KCl^36^) and *c* is the concentration of the redox probe (*c* = 1.0 × 10^–6^ mol cm^–3^). By comparing the experimental slopes of the *I*_p_ vs. *v* ^1/2^ curves (Figure 8b and d) with the theoretical slope of the Randles-Ševčík equation (eq 1), the electroactive area for each electrode was predicted. The geometric area of the working electrodes evaluated was the same and equal to 0.196 cm^2^. The electroactive surface areas found for CB-PAD and 5GP-CB-PAD were 0.235 cm^2^ and 0.239 cm^2^, respectively.

An important kinetic parameter predicted for the designed electrodes evaluated was the *k* ^0^ constant. The Nicholson method was used for this, which can be applied to calculate heterogeneous electron transfer rate constants for quasi-reversible systems controlled by diffusion.^37^ eq 2 shows the relationship for obtaining *k* ^0^

2 $$\Psi =k^{0}{\left[\pi DnvF/\left(RT\right)\right]}^{-1/2}$$

where Ψ is a kinetic parameter, and the other parameters have their usual meanings. The Ψ values were obtained using eq 3 proposed by Lavagnini et al.^38^

3 $$\Psi =\left(-0.6288+0.0021\Delta E_{p}\right)/\left(1-0.017\Delta E_{p}\right)$$

Given the calculated Ψ values, the constant *k* ^0^ follows from the slope of the Ψ vs 32.79 *v* ^–1/2^ plot obtained for each electrode (Figure S3). The factor 32.79 is equivalent to the term [πD*n* F/(RT)]^−1/2^, calculated considering *D* = 7.6 × 10^–6^ cm^2^ s^–1^, *F* = 96485C mol^–1^, *R* = 8.314 J K^–1^ mol^–1^ and *T* = 298.15 K. The linear regression equations gave a *k* ^0^ value equal to (3.5 ± 0.3) × 10^–3^ cm s^–1^ (CB-PAD) and (6.0 ± 0.3) × 10^–3^ cm s^–1^ (5GP-CB-PAD), respectively. These results indicate that the kinetic constant almost doubled with the addition of graphene to the ink formulation. The improved charge transfer kinetics resulting from the combination of carbon black and graphene in the composition of conductive inks for the manufacture of SPEs can be understood as a synergistic effect, in which graphene sheets can be spaced by carbon black nanoparticles, while the graphene network connects CB nanoparticles, leading to a gain in conductivity and greater electron transfer capacity. As observed by Vignesh et al.,^39^ in their study of a sensor modified with a graphene surface for detecting BPA.

### 3.3 Electrochemical Detection of BPA

Subsequently, the analytical performance of the proposed 5GP-CB-PAD as an electrochemical sensor was tested toward the determination of the BPA endocrine disruptor as a proof of concept. Thus, a single anodic peak was verified at + 354 mV, which suggests that BPA underwent irreversible oxidation (Figure 9). Studies dedicated to the irreversible oxidation mechanism of BPA demonstrate a sequence of steps involving the diffusion of BPA molecules from the bulk of the solution to the inner Helmholtz plane, with adsorption of BPA molecules on the electrode’ surface and sequential electron transfer, forming a partially oxidized species in a first step and then the fully oxidized product, with the total transfer of two electrons in a pH-dependent process.^40^ The inset of Figure 9 shows the global possible BPA oxidation mechanism,^41^ where the phenol groups present in the BPA structure are oxidized to carbonyl groups (C=O). The obtained voltammetric profile is in line with what is expected for this analyte, and other authors have also reported an irreversible redox process for BPA.^42,43^

**Figure 9:**
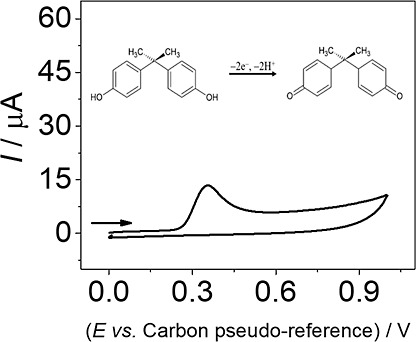
Cyclic voltammogram obtained in 0.2 mol L^–1^ phosphate buffer solution (pH 8.0) containing 5.0 × 10^–4^ mol L^–1^ BPA using the 5GP-CB-PAD. *v* = 50 mV s^–1^. BPA preaccumulation: application of 0.0 V for 120 s. Inset: possible BPA oxidation mechanism.

Optimization studies of the analysis parameters were carried out to find the best conditions for the voltammetric detection and subsequent quantification of BPA. SWV measurements were also carried out since this technique is sensitive and can more adequately discriminate between the faradaic and capacitive currents. Furthermore, it is worth emphasizing that all optimization tests were performed in triplicate, allowing for an evaluation of the voltammetric profile of the BPA response and the variability of the analytical signal across the different levels evaluated.

The effect of pH of the supporting electrolyte solution was initially studied. Figure 10a,b show the obtained cyclic voltammograms and square-wave voltammograms toward BPA in phosphate buffer solutions at different pHs (range of 2–10) using the proposed 5GP-CB-PAD sensor.

**Figure 10:**
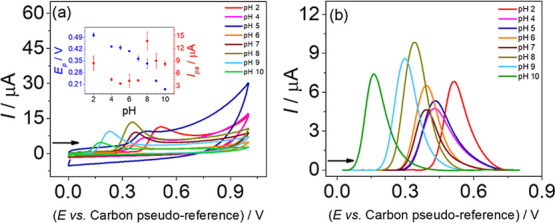
(a) Cyclic voltammograms and (b) square-wave voltammograms obtained in 0.2 mol L^–1^ phosphate buffer solutions at different pHs (2.0 to 10) containing 5.0 × 10^–4^ mol L^–1^ BPA using the 5GP-CB-PAD. *v* = 50 mV s^–1^ (CV). SWV parameters: *f* = 25 Hz, *a* = 20 mV, and Δ*E*_s_ = 5 mV. BPA preaccumulation: application of 0.0 V for 120 s.

By using both voltammetric techniques, it was clear that pH played a pronounced effect on the irreversible oxidation of BPA. From the CVs of Figure 10a, plots of *E*_pa_ vs pH and *I*_pa_ vs pH were constructed (See the inset in Figure 10a). By looking at the *I*_pa_ vs pH curve, it was found that the highest peak current analytical signal for BPA was recorded at pH 8.0. At pHs above 8.0, there was a drop in the measured current. Additionally, the increase in pH resulted in a displacement of *E*_pa_ to less positive values, which in the literature is discussed as a typical characteristic of redox processes where prior protonation of the analyzed species occurs.^41,43^ The loss of BPA voltammetric response to solutions with pH above 8.0 can be rationalized as follows: the p*K*_a_ of BPA is ∼9.76.^41^ At pH > p*K*_a_, BPA is in its anionic form (partially or deprotonated species), which can lead to repulsion forces between the anionic species and the π-electrons and oxygen-functional groups of graphene and carbon black. Thus, at pH 8.0 there is a predominance of the fully protonated species, which can adsorb more effectively on the sensor surface. The square-wave voltammograms of Figure 10b agree with the data recorded by cyclic voltammetry, i.e., a maximum anodic peak current signal was registered at pH 8.0. Other sensors already reported in the literature for detecting BPA also describe pH 8.0 as the optimum pH for detecting this analyte.^44,45^ Consequently, pH 8.0 of the supporting electrolyte was chosen for further assays.

Preaccumulation of BPA on the sensor’s surface can provide enhanced sensitivity and subsequently allow for lower limits of detection. For this reason, the optimum accumulation time of BPA on the surface of the 5GP-CB-PAD was investigated. It has been previously reported in the literature that applying positive or negative potentials for BPA accumulation on electrochemical sensors resulted in an analytical signal statistically similar to that obtained at 0.0 V; for this reason, it was decided to set the applied potential in this study at 0.0 V.^45^ Figure S4a shows the square-wave voltammograms obtained at different BPA preaccumulation times (30–180 s). It can be seen that the highest intensity signal was verified at a preaccumulation time of 30 s, and at longer times there was a significant drop in the measured signal. This preaccumulation time is shorter than that of other sensors described in the literature^44–46^ which demonstrates the potential of the sensor proposed here for fast analysis of BPA. The short preaccumulation time of BPA on the surface of the 5GP-CB-PAD ensures that the sensor operates at its maximum efficiency, without being affected by the absorption of components of the measured samples, which is characterized as another advantage, and which justifies the chosen accumulation time of 30 s.

Finally, the technical parameters of SWV were also subjected to optimization, including frequency (*f*), amplitude (*a*), and potential increment (Δ*E*_p_).

The set of results from these optimizations can be found in Figure S4b–d. The tests were carried out univariately and sequentially, initially assessing the effect of frequency (Figure S4b), then the effect of amplitude (Figure S4c), and finally the effect of potential increment (Figure S4d). For the frequency and amplitude parameters, the values chosen were those that resulted in the highest computed analytical signal, i.e., 25 Hz and 100 mV, respectively. Although the 5 mV potential increment provided the highest detectable analytical signal for BPA, a higher standard deviation between measurements was verified in this condition, which is why the 3 mV step value was chosen.

Once the main parameters that could influence the detection and quantification of BPA had been optimized, the analytical curve was constructed. The curve was constructed by measuring one point per electrode, and the measurements were taken in triplicate. Figure 11a shows the SWVs obtained in the presence of different BPA concentrations. Thus, the 5GP-CB-PAD sensor proved to be sensitive toward BPA, where it was possible to see an increase in the analytical signal as the concentration of BPA increased. Indeed, a linear relationship was verified between anodic peak current and BPA concentration, as displayed by the analytical curve of Figure 11b (*I*_pa_ vs c­(BPA) curve). The analytical curve was linear in the concentration range of 5.0 × 10^–6^ to 5.0 × 10^–4^ mol L^–1^ of BPA, according to the linear regression equation: *I*_pa_ (*A*) = 1.208 × 10^–6^ + 0.055 c­(BPA) (mol L^–1^), *R* ^2^ = 0.993. The limits of detection (LOD) and quantification (LOQ) were calculated as 3.3 times (or 10 times for LOQ) the standard deviation of 10 measurements of the blank (i.e., only supporting electrolyte), divided by the sensitivity (slope of the analytical curve). Thus, the values found were LOD = 1.7 × 10^–6^ mol L^–1^ and LOQ = 5.1 × 10^–6^ mol L^–1^, respectively.

**Figure 11:**
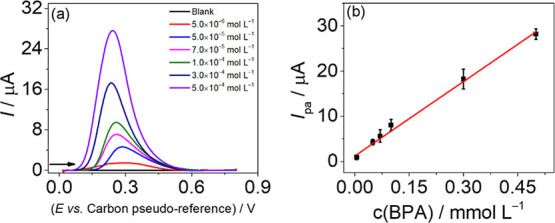
(a) Square-wave voltammograms obtained in 0.2 mol L^–1^ phosphate buffer solution (pH = 8.0) containing different BPA concentrations using the 5GP-CB-PAD sensor. SWV parameters: *f* = 25 Hz, *a* = 100 mV and Δ*E*_s_ = 3 mV. (b) Analytical curve recorded from the SWV data using the *I*_pa_ vs c­(BPA) relationship. Measurements carried out in triplicate.

To evaluate the repeatability of the implemented PAD manufacturing procedure, different sensors (*n* = 5) were used, and SWV measurements were performed in 0.2 mol L^–1^ phosphate buffer (pH 8.0), in the presence of 8.5 × 10^–5^ mol L^–1^ BPA, under optimized analytical conditions. A relative standard deviation (RSD %) of 9.73% was obtained for the peak currents recorded among the five different PADs. With the result obtained, it is possible to demonstrate that even though the application of the conductive ink was manual, the methodology followed allowed for the production of the 5GP-CB-PAD with good repeatability for quantitative analysis of BPA. A stability study of the 5GP-CB-PAD sensor was also conducted (*n* = 4) to analyze BPA under optimized analytical conditions. The tests were performed at time points of 0, 1, 5, and 10 days from the day the sensor was prepared. The RSD (%) obtained was 7.51%, indicating good stability of the analytical signal generated.

To evaluate the performance of the 5GP-CB-PAD assay in determining BPA in the presence of other molecules, an interference study was conducted. For clarification purposes, uric acid, an organic molecule, and the ion pairs CO_3_^2–^/NO_3_^–^ and Ca^2+^/Mg^2+^ were evaluated. The tests were conducted at a 1:1 ratio of BPA to potential interfering species under optimized analytical conditions. From Figure S5 and Table S1, it can be observed that the analytical signal for BPA varied by less than 2% in the presence of uric acid and the Ca^2+^/Mg^2+^ pair, demonstrating the sensor’s selectivity for BPA in the presence of these interferents. However, interference exceeding 10% was observed in the presence of the CO_3_^2–^/NO_3_^–^ pair. Thus, further studies should be conducted to minimize this interference.

Many of the previously reported studies on the voltammetric sensing of BPA include application studies on river water samples,^47–50^ canned food,^51,52^ plastic waste,^53,54^ and milk samples,^55,56^ to demonstrate the potential use/or limitation of the proposed sensors. In this work, we conducted recovery studies of added BPA concentrations in tap water samples collected at the LSNano UFSCar (Araras Campus, SP, coordinates: −22.314949898743244, −47.38043727234557) and, in green corn canning water as a food sample matrices. Table 1 shows the obtained recovery results.

**Table 1:** Results of BPA Recovery Studies at Different Matrices Samples Using SWV and the Proposed 5GP-CB-PAD Sensor

	c(BPA)/mol L^–1^		
sample	added	found	recovery (%)^a^
tap water	6.0 × 10^–5^	(6.46 ± 0.01) × 10^–5^	107.67
	2.0 × 10^–4^	(2.26 ± 0.01) × 10^–4^	113.00
corn canning water	6.0 × 10^–5^	(5.99 ± 0.01) × 10^–5^	99.83
	2.0 × 10^–4^	(1.69 ± 0.04) × 10^–4^	84.50

a Recovery (%) = (c­(BPA) _Found_/c­(BPA)_Added_) × 100%, *n* = 4.

No BPA was detected in the samples analyzed, either because it was absent or because the concentration was below the linear range of the designed sensor. Therefore, the samples were fortified with BPA and analyzed for recovery percentage. As can be seen from Table 1, recovery percentages higher than 84% were obtained. For the tap water sample, where the values found were above the expected values, it can be suggested that salts and other species present in the sample caused some interference in the analysis. For future analysis of samples with this characteristic, the standard addition method can be used, which will eliminate any possible matrix effects. In this study, 5GP-CB-PAD was used without any treatment or cleaning of the samples. Thus, the sensor showed its applicability and functionality in different samples in which BPA can be found.

Table 2 compares the performance of sensors already reported in the literature for analyzing BPA with the results found for the proposed work.

**Table 2:** Comparison of BPA Detection Performance by Sensors Already Reported in the Literature

sensor	substrate	technical	working range (μmol L^–1^)	LOD (μmol L^–1^)	ref
Gr-AuNP-ext/GCE	—	DPV	0.05–10.0	0.015	^57^
CdTe@QDs-FG-PEDOT:PSS/SPE	polyester sheet	SWV	0.020–1.0; 1.0–9.0	0.0034	^58^
WS_2_–Fe_3_O_4‑_rGO/LabSPE	polycarbonate	DPV	0.05–50	0.03	^59^
PANI/Au/PET	polyethylene terephthalate (PET, thickness ∼125 μm)	CV	0.05–5	1.06 × 10 ^**–**3^	^20^
GO/FeNps/GCE	—	SWV	15.0–120.0	12.05	^59^
MIP/CoNi-MOF/PANI/RGO/GCE	—	DPV	0.001–0.5	3.6 × 10^–4^	^60^
PPB700/SPCE	—	CV	50–300	77	^61^
PIB-SEFG/SPE	rigid transparent polyvinyl chloride sheets (PVC)	SWV	2.5–200	1.7	^62^
3NBC/GCE	—	DPV	0.005–25	1.75	^63^
MnO/CB	commercial portable sensor	DPV	0.4–15.6; 15.6–85.6	0.12	^64^
**5GP-CB-PAD**	sulfite paper (75 g m^–2^)	**SWV**	5.1–500	1.7	**this work**

**3NBC/GCE**: bifunctional biochar-modified glassy carbon electrode doped with nitrogen; **CdTe@QDs-FG-PEDOT/PSS/SPE**: electrode screen-printed with functionalized graphene ink, cadmium telluride quantum dots and polymer; **CoNiP@rGO/GCE**: glassy carbon electrode modified by nanoparticles of bimetallic cobalt-nickel phosphide (CoNiP) supported on reduced graphene oxide (rGO); **CuL­(CH_3_COO)_2_/SPE**: screen-printed electrode of amide-based macrocyclic complex (nanostructured complex of copper acetate with macrocyclic ligand (CuL­(CH_3_COO)_2_)); **GO/FeNps**: glassy carbon electrode modified by graphene oxide nanostructures and anchored iron nanoparticles; **Gr-AuNP-ext/GCE**: glassy carbon electrode modified by graphene and gold nanoparticles, the latter reduced and stabilized by guava extract; **MIP/CoNi-MOF/PANI/RGO/GCE**: carbon glass electrode modified by molecular imprinting polymers, reduced graphene oxide, polyaniline, and cobalt-nickel metal–organic framework; **MnO/CB**: commercial electrode modified with carbon black and manganese oxide; **PANI/Au/PET**: screen-printed electrode whose working electrode was a gold film with deposition of unlabeled polyaniline (PANI); **PIB-SEFG/SPE**: electrode screen-printed with a conductive ink based on 10% graphene nanoplates, 40% nanographite powder and 50% glass varnish (m/m); **PPb700/SPCE**: carbon electrode printed on fabric modified by biochar derived from pineapple peel; **Pt@SWCNTs-Ti**_3_C_2_ **-rGO/SPCE**: electrode screen-printed with a combination ink of Pt nanoparticles modified with single-walled carbon nanotubes (Pt@SWCNTs), MXene (Ti_3_C_2_) and graphene oxide (GO); **WS_2_–Fe_3_O_4_**_‑_ **rGO/LabSPE**: printed electrode modified with reduced graphene oxide, magnetite, and tungsten disulfide.

Recent studies described in the literature on the electrochemical detection of BPA show the use of glassy carbon electrodes and screen-printed electrodes for the analysis of the monomer. To improve the sensitivity and/or selectivity of the electrode, modifications have been made to them, such as the incorporation of metallic nanoparticles,^44,50^ carbon materials such as carbon black,^48^ graphite, graphene and derivatives^41,46,62,65^ and conductive polymers.^20,58,66,67^

From the literature review presented in Table 2, we can see the variety of screen-printed electrodes (SPEs) and PADs already produced for the analysis of BPA. Among them, it can be seen that the sensor proposed in this work provided similar and/or better analytical parameters than the sensors designed using glassy carbon electrodes (GCE) in their detection system. This comparison indicates the possibility of using SPEs in the development of (bio)­sensors without affecting the detection of BPA, with the advantage of ease of production and lower cost per sensor. Although the LOD obtained was comparable to or higher than that of other sensors previously reported in the literature, the 5GP-CB-PAD did not require costly steps or higher-cost selectivity elements for its construction. Therefore, it can be argued that the 5GP-CB-PAD proved to be competitive with sensors already reported in the literature, which justifies the attempt to apply this sensor in a valid manner.

## 4 Conclusion

This article reports on the successful fabrication of an analytical device based on sulfite and shellac paper and conductive carbon black and graphene ink. The combination of an easily accessible and cost-effective paper and shellac, a good binder that dries quickly, enabled the manual manufacture of sensitive electrochemical sensors with good repeatability and reproducibility and sensitivity for the determination of bisphenol A (BPA). The addition of graphene to the paint did not increase the electroactive area of the sensor, but almost doubled the *k* ^0^. A wide linear working range of 5.0 × 10^–6^ to 5.0 × 10^–4^ mol L^–1^ was recorded for BPA, as well as LOD and LOQ of 1.7 × 10^–6^ mol L^–1^ and 5.1 × 10^–6^ mol L^–1^, respectively. The simplicity, as well as the possibility of adjusting the sensor construction steps according to the structure, equipment and financial resources of each laboratory, facilitates the implementation and use of PADs in research and in the field. In addition, the sustainability of the paper substrate, its biocompatibility and its proper disposal consolidate the proposed sensor as a viable alternative for the development of new portable and disposable sensors applicable to the detection of bisphenol A monomer. The future use of 5GP-CB-PAD in routine analysis is dependent on increasing selectivity for BPA, either by adding BPA-selective biomolecules and/or cleaning more complex samples before determining the monomer.

## Supplementary Material



## Notes

Figure S1–EDS mapping for different materials (PDF); Figure S2–images of the wettability analysis by contact angle (PDF); Figure S3–plots of Ψ vs 32.79 *v* ^–1/2^ obtained from cyclic voltammetry data for the FCN redox probe using CB-PAD and 5GP-CB-PAD (PDF); Figure S4–square-wave voltammograms obtained in 0.2 mol L^–1^ phosphate buffer solutions (pH = 8.0) containing 5.0 × 10^–4^ mol L^–1^ BPA using the 5GP-CB-PAD during different optimizations (PDF); Figure S5–bar chart of the analytical signal obtained from the 5GP-CB-PAD sensor for the analysis of BPA solution and BPA in the presence of the interferents uric acid and ion pairs CO_3_^2–^/NO_3_^–^ and Ca^2+^/Mg^2+^. Studies conducted under optimized analytical conditions (PDF); Table S1–study of possible interferents on the voltammetric sensing of BPA (PDF) (PDF)

## Notes

Alexsandra Dias da Silva–Department of Chemistry, Federal University of Viçosa, 36570-900, Viçosa, MG, Brazil, Jéssica Rocha Camargo–Laboratory of Sensors, Nanomedicine, and Nanostructured Materials, Federal University of São Carlos, 13600-970, Araras, SP, Brazil, Rodrigo Silva de Oliveira–Department of Chemistry, Federal University of Viçosa, 36570-900, Viçosa, MG, Brazil, Bruno Campos Janegitz–Laboratory of Sensors, Nanomedicine, and Nanostructured Materials, Federal University of São Carlos, 13600-970, Araras, SP, Brazil, Tiago Almeida Silva–Department of Chemistry, Federal University of Viçosa, 36570-900, Viçosa, MG, Brazil
