# Supplementary material for: Eco-Friendly Paper-Based Electrochemical Device Manufactured with Low-Cost School Supplies and Carbon Black-Graphene Ink for Determination of Endocrine Disruptor in Food
Source: ACS Omega. 2026 Jun 30;11(27):40173–83. doi: 10.1021/acsomega.6c02401 (PMC13382684; doi:10.1021/acsomega.6c02401)
Supplement: Supplementary file 1 [file ao6c02401_si_001.pdf]

Supplementary Material for the Paper

ECO-FRIENDLY PAPER-BASED ELECTROCHEMICAL DEVICE MANUFACTURED WITH LOW-COST SCHOOL SUPPLIES AND CARBON BLACK-GRAPHENE INK FOR DETERMINATION OF ENDOCRINE DISRUPTOR IN FOOD

Alexsandra Dias da Silva[a], *Jéssica Rocha Camargo*[b], *Rodrigo Silva de Oliveira*[a], *Bruno Campos Janegitz*[b] *and Tiago Almeida Silva*[a]*

[a]Department of Chemistry, Federal University of Viçosa, 36570-900, Viçosa, MG, Brazil.

[b]Laboratory of Sensors, Nanomedicine, and Nanostructured Materials, Federal University of São Carlos, 13600-970, Araras, SP, Brazil.

*Email: tiago.a.silva@ufv.br

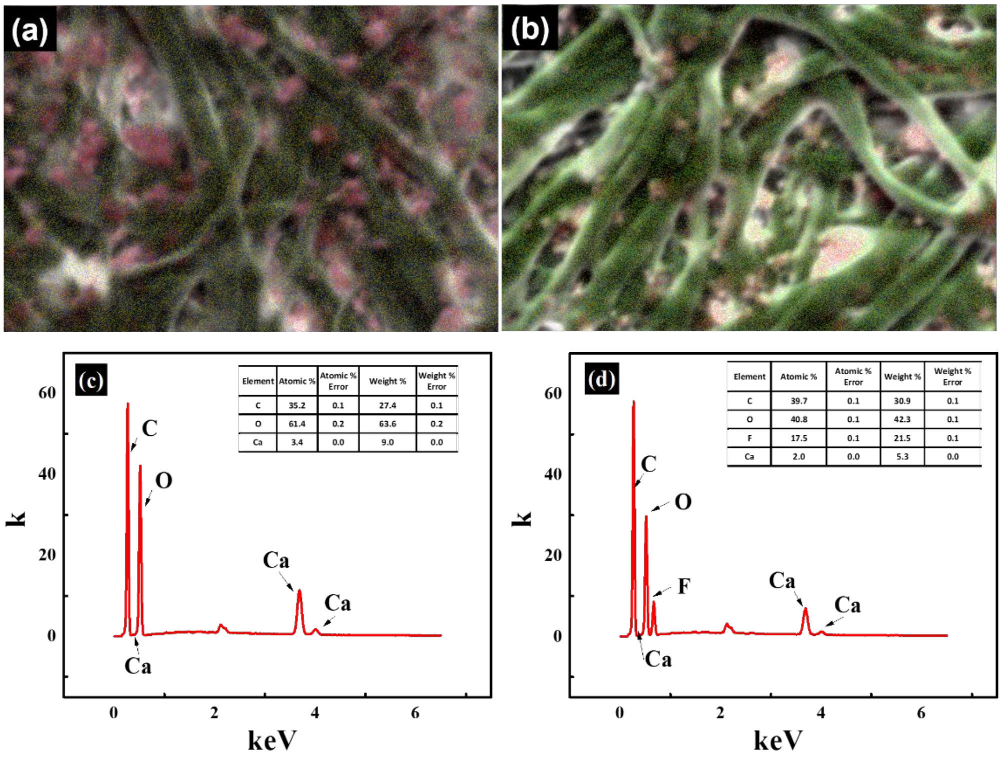

Figure S1. EDS mapping for **(a)** sulphite paper without waterproofing agent and **(b)** sulphite paper with waterproofing applied. EDS for **(c)** sulphite paper without waterproofing agent and **(d)** sulphite paper with waterproofing applied.

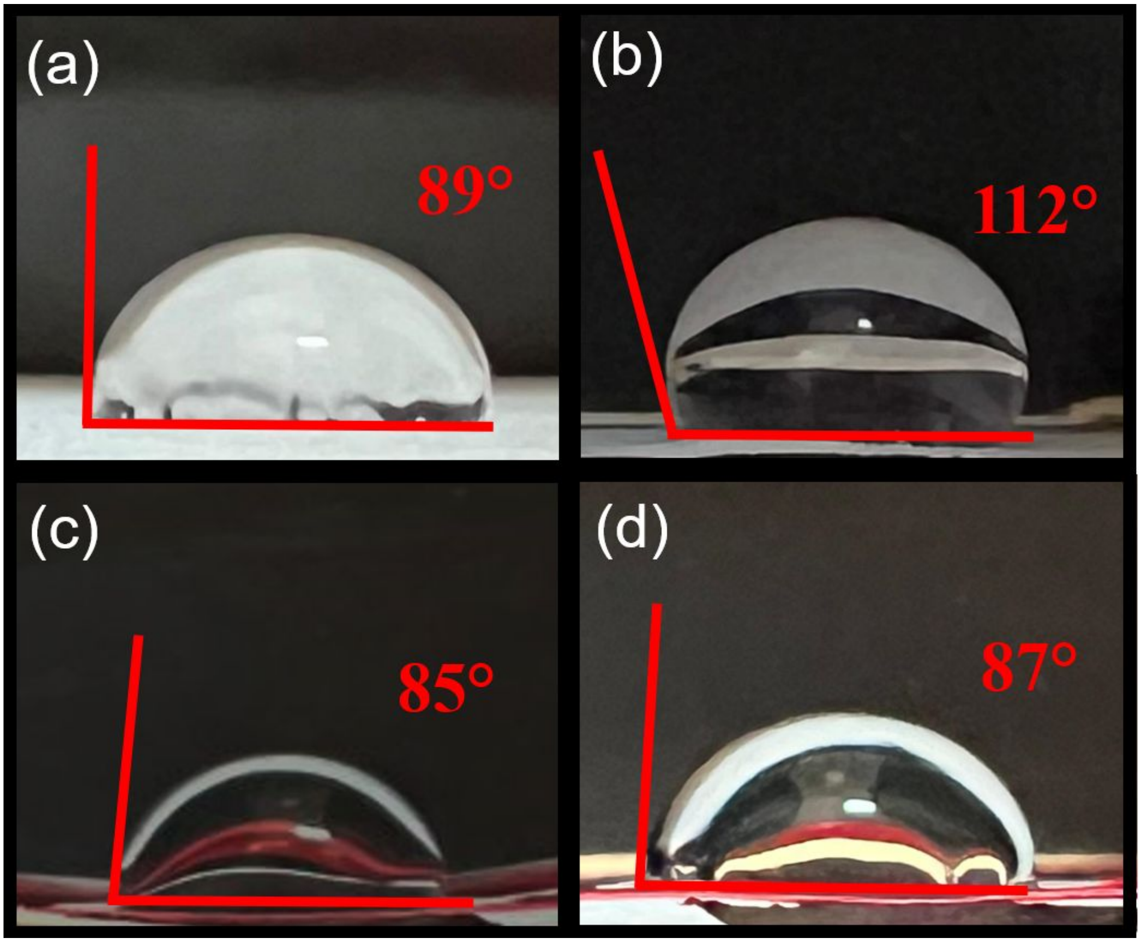

Figure S2. Images of the wettability analysis by contact angle in the **(a)** absence and **(b)** presence of waterproofing on the paper, as well as on the working electrodes of the **(c)** CB-PAD and **(d)** 5GP-CB-PAD devices.

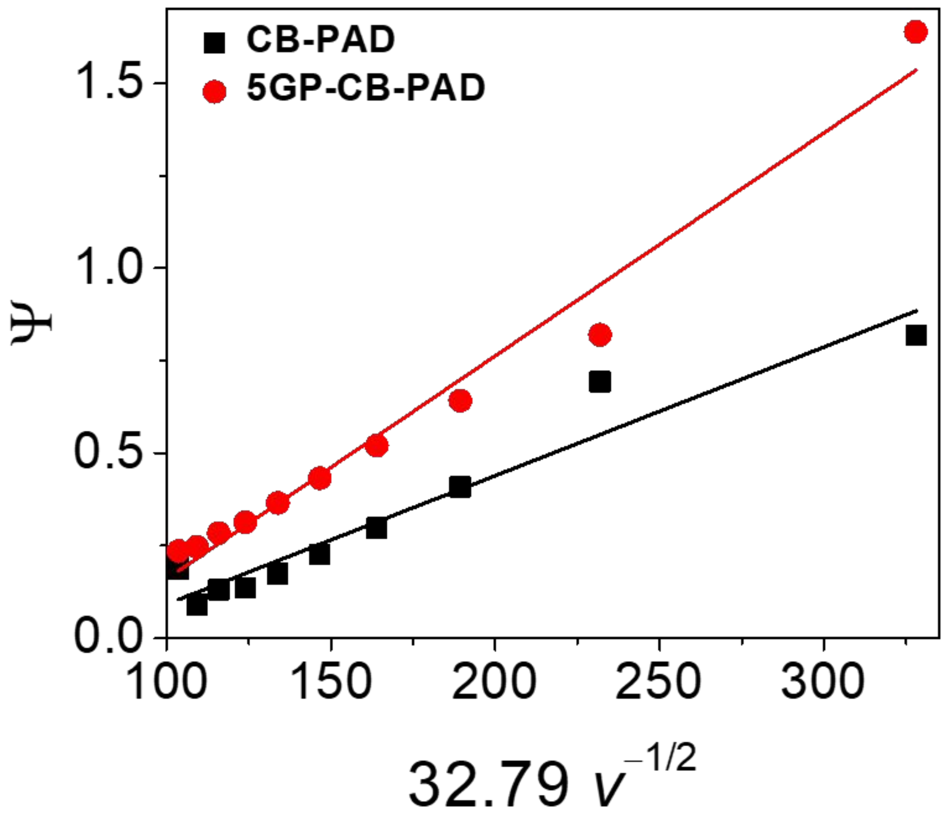

Figure S3. Plots of Ψ *vs*. 32.79 $v^{-1/2}$ obtained from cyclic voltammetry data for the FCN redox probe using CB-PAD and 5GP-CB-PAD.

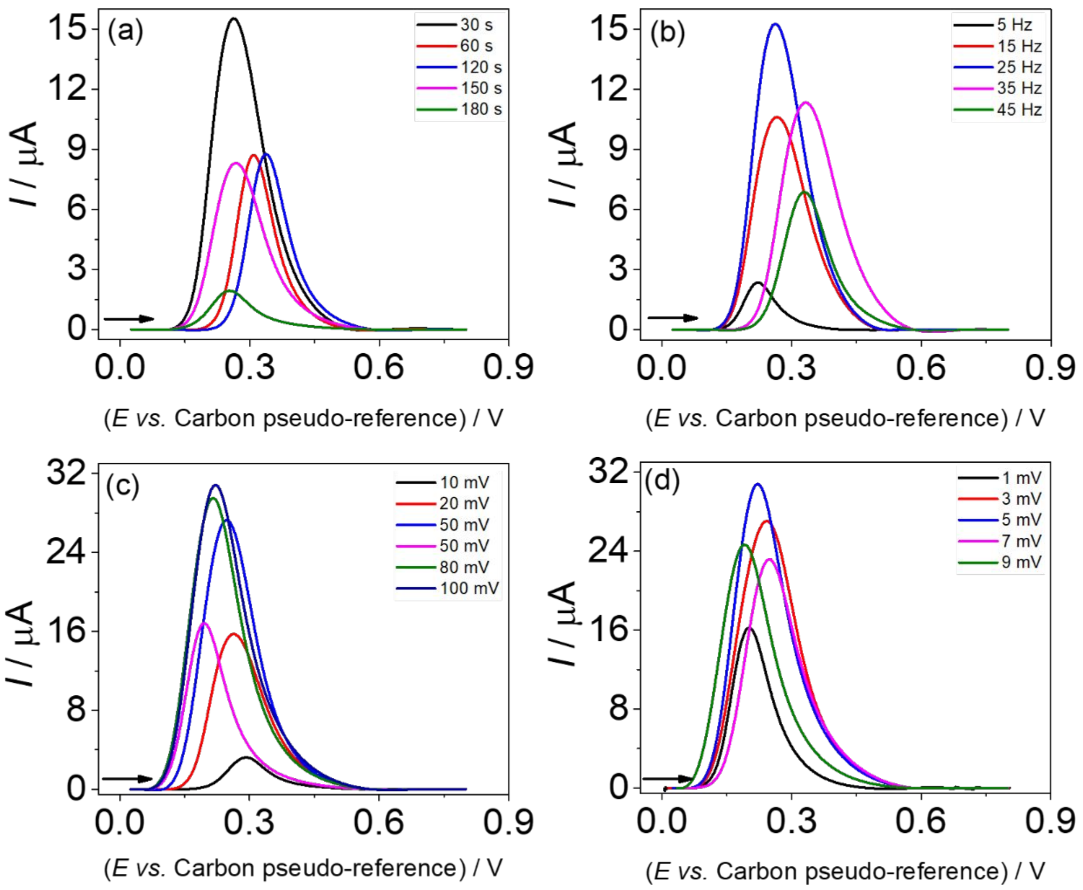

Figure S4. Square-wave voltammograms obtained in 0.2 mol L^{-1} phosphate buffer solutions (pH = 8.0) containing 5.0×10^{-4} mol L^{-1} BPA using the 5GP-CB-PAD during optimization of **(a)** BPA pre-accumulation time (30-180 s) at 0.0 V, **(b)** frequency (5-45 Hz), **(c)** amplitude (10-100 mV) and **(d)** step potential (1-9 mV).

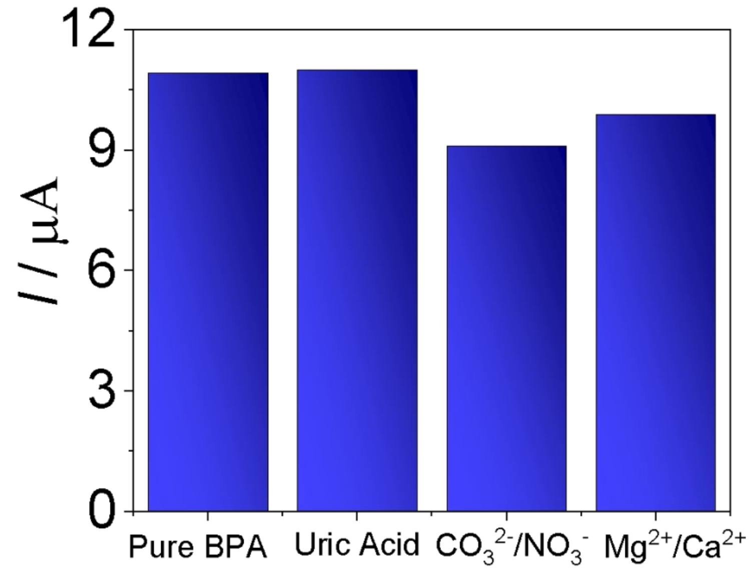

Figure S5. Bar chart of the analytical signal obtained from the 5GP-CB-PAD sensor for the analysis of BPA solution and BPA in the presence of the interferents uric acid and ion pairs CO_3^{2-} / NO_3^- and Ca^{2+} / Mg^{2+}. Studies conducted under optimized analytical conditions.

Table S1. Study of possible interferents on the voltammetric sensing of BPA

**Interferent**	**Molar ratio / c(BPA):c(Interferent)**	**Change in I_{pa} / %**
Uric Acid	1:1	+0.73
CO_3^{2-} / NO_3^-	1:1	−16.74
Ca^{2+} / Mg^{2+}	1:1	−1.38
